# Antitetanic Serum as a Prophylactic

**Published:** 1904-08

**Authors:** 


### Antitetanic Serum as a Prophylactic.

Dr. Joseph McFarland, (*Journal American Medical Association,* July 4, 1903,) reports the results of a series of observations upon eight hundred horses which illustrate the value of antitetanic serum as a prophylactic agent. During a period of four years there had been a death-rate of 10 per cent. from tetanus, in spite of all precautions. A systematic immunisation with antitetanic serum was then begun. Injections of 10 to 25 c.c. of serum were given every three months. As a result, the death-rate from tetanus rapidly decreased, and in the second year had been reduced to less than 1 per cent. The author believes that the practical conclusions to be drawn from these observations may be applied to the human subject. He thinks that antitetanic serum should be given as a prophylactic measure in all cases of suspicious wounds that are likely to be followed by tetanus. Experiments made on guinea pigs by the author demonstrated that the dried serum fully protects inoculated animals. In further elaboration of this subject we quote the following from the *Virginia Medical Semi-Monthly,* which says: an editorial in the *New York Medical Journal,* March 26, 1904, remarks that "the present drift of opinion seems to be to the effect that tetanus antitoxin, while probably of considerable prophylactic efficacy, is of little use as a curative agent."

Recent experience in the immediate topical employment of this antitoxin in cases of toy-pistol injuries appears to support the trust in its prophylactic value.

From such experiences as above referred to it has been proposed to immediately inject antitetanic serum in every case of traumatism of a suspicious character—hoping in this manner to prevent the subsequent development of tetanus. It has been suggested to inject prophylactically all new-born infants in certain sections of Europe in which trismus neonatorum prevails.

The serum is harmless to man, and may be given hypodermatically as the other serums. Nocard recommends that a first injection of ten cubic centimeters should be made in adults as soon as possible after traumatism. A second injection should follow in from twelve to fifteen days. The use of antitetanic serum in no way precludes the employment of spinal antispasmodic remedies, as chloral, bromides, morphine, eserine, and the like.

